# Semantic-Guided Multi-Level Collaborative Fusion Network for Visible and Infrared Images

**DOI:** 10.3390/s26092577

**Published:** 2026-04-22

**Authors:** Lijun Yuan, Chuanjiang Xie, Ming Yang, Xiaoguang Tu, Qiqin Li, Xinyu Zhu

**Affiliations:** 1 College of Aviation Electronics and Electrical, Civil Aviation Flight University of China, Guanghan 618307, China; yuanlijun33320@163.com (L.Y.); cj_x_edu@163.com (C.X.); txg198955@163.com (X.T.); fredzhe4455@163.com (Q.L.); 2 School of Optoelectronic Science and Engineering, University of Electronic Science and Technology of China, Chengdu 610054, China; 3 Sichuan Province Engineering Technology Research Center of General Aircraft Maintenance, Civil Aviation Flight University of China, Guanghan 618307, China

**Keywords:** semantic prior, cross-attention transformer, feature calibration, multi-level interaction, cross-modal fusion, object detection

## Abstract

**Highlights:**

**What are the main findings?**

- We propose a semantic-guided collaborative network for visible and infrared image fusion that maintains semantic guidance throughout the fusion process, generating semantically consistent and detail-preserving fused representations.
- Stage-wise cross-modal calibration and a three-level interaction strategy are constructed to strengthen early-stage information exchange and progressively inject semantic priors into cross-modal fusion and intra-modal feature learning.

**What are the implications of the main findings?**

- Preserving global semantic context during up sampling is essential to mitigate semantic dilution and guide task-oriented fusion model design.
- This study suggests that semantic-guided collaborative fusion represents a practical direction for enhancing perceptual quality and downstream object detection.

**Abstract:**

The paramount value of image fusion is manifested in effectively enhancing downstream tasks. However, compatibility with subsequent tasks is compromised due to the semantic deficiency of fusion representations generated by current approaches. To mitigate this limitation, a semantic-guided multi-level collaborative fusion network is proposed, termed DSIFuse. By leveraging semantic priors and global context extracted from auxiliary segmentation branches, a multi-level interaction space is constructed to explicitly refine cross-modal features. Specifically, a cross-modal feature correction mechanism is designed to enhance semantic alignment by injecting complementary visible–infrared information at each layer, while a three-level interaction strategy gradually integrates unimodal features and semantic maps to generate semantically enriched representations. To mitigate semantic information loss during image reconstruction, a semantic compensation block is employed, incorporating interactive representations from prior layers and global semantic maps into the multi-scale decoder. Finally, the overall loss integrates semantic supervision, gradient, and intensity loss. Experiments conducted on public datasets indicate that clear fusion images are generated by DSIFuse, with improved structural consistency and reduced artifacts. Under a unified benchmark, the fused representations subsequently yield improved performance in downstream object detection tasks.

## 1. Introduction

Multispectral sensors acquire complementary information from diverse imaging modalities, providing a richer basis for subsequent fusion and analysis [1]. Infrared sensors excel at thermal radiation imaging under extreme conditions but lack textural details. Conversely, visible sensors preserve abundant structural and textural information, yet their performance deteriorates in low-illumination and harsh environments. By integrating the complementary strengths of these two modalities, infrared–visible image fusion is expected to generate more informative representations for downstream tasks such as object detection, scene understanding, and multimodal tracking [2,3,4].

Despite achieving substantial progress, a significant limitation remains in existing fusion research. Fused images are still evaluated primarily from the perspective of human visual perception, whereas semantic completeness and compatibility with downstream machine vision tasks are often insufficiently considered. For high-level vision applications, a fused representation should preserve not only prominent visual content but also semantically consistent cross-modal cues that facilitate reliable inference. When semantic guidance is insufficient during intermediate feature learning, fused images frequently exhibit incomplete object semantics, blurred boundaries, and unstable cross-modal correspondence, ultimately constraining their applicability in downstream tasks.

**Figure 1:**
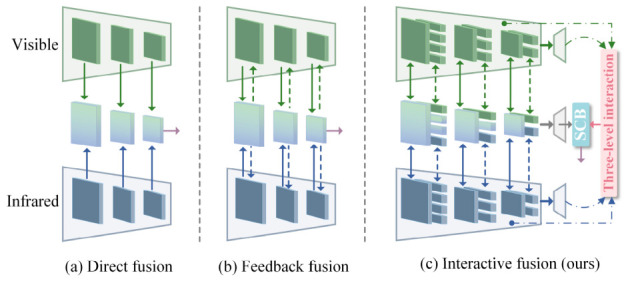
Workflow comparison: existing fusion paradigm versus the proposed paradigm. Green represents the visible branch, blue represents the infrared branch.

In response to these challenges, we propose a semantic-guided multi-level collaborative fusion network for infrared and visible image fusion, abbreviated as DSIFuse. Our method is meticulously engineered to enhance semantic consistency, retain intricate details, and improve compatibility with downstream tasks by employing multi-level collaboration. Specifically, this work presents a hybrid collaborative fusion framework that strategically combines the advantages of direct and feedback fusion to enable hierarchical cross- and intra-modal interactions, Figure 1c. Semantic information loss is effectively mitigated by introducing semantic priors via a three-stage interaction and applying semantic compensation during the decoding phase. This comprehensive strategy ultimately yields semantically consistent and detail-rich fused representations, which are more compatible with object detection in our evaluated benchmark setting. Comprehensive experiments conducted on public datasets reveal that DSIFuse attains competitive performance in fusion metrics while also showing competitive object detection results under a unified benchmark. These findings indicate that a unified semantic-guided collaborative design represents a promising and effective approach for task-oriented visible–infrared image fusion.

## 2. Related Works

### 2.1. Semantic-Guided Image Fusion

Existing multimodal semantic segmentation methods for visible–infrared modalities are broadly categorized into two principal paradigms (as depicted in Figure 1): direct fusion [5,6,7] and feedback fusion [8,9,10]. In the direct fusion paradigm Figure 1a, two separate backbone networks are employed to extract multi-level features from each modality. The extracted features are subsequently integrated at various levels via a fusion module that operates independently of the backbone networks. However, such approaches generally exhibit the following limitations: they typically utilize a uniform fusion strategy to process features from different levels, thereby overlooking the inherent semantic evolution of features from low to high levels. Simultaneously, the absence of information exchange between fusion layers often leads to an isolated, level-by-level fusion pattern. For instance, while RTFNet [7] targets real-time performance, supervision signals are applied solely at its final output layer. This insufficient supervision during intermediate feature extraction and fusion processes often leads to segmentation outputs characterized by blurred boundaries and category confusion. Similarly, EFNet [11] excels in designing efficient fusion modules, rendering it suitable for applications with stringent real-time requirements. CrossNet [12] enhances feature integration accuracy through intricate strategies. However, both these approaches share core issues: rigid fusion mechanisms and isolated information flow between fusion layers.

In contrast, feedback fusion Figure 1b introduces an interaction mechanism that augments direct fusion by feeding backbone-independent fused features back into their corresponding branches, thereby establishing multi-level interactions between the encoder and fusion layers. As representative models of the feedback fusion paradigm, RFNet [13] and BiFNet [14] introduce recurrent or bidirectional interaction mechanisms, enhancing feature exchange compared to direct fusion approaches. Nevertheless, these methods still contend with challenges, including information bottlenecks, suboptimal utilization of modal complementarity, and sparse supervision signals. While such methods indeed enhance feature interactions, the inherent feedback loops may inadvertently lead to information bottlenecks, where shallow layers rich in fine-grained detailed features struggle to be recursively integrated into deeper networks to form comprehensive high-level semantics. Therefore, the complementarity between modalities also fails to be effectively leveraged during multi-level interactions. Furthermore, both aforementioned paradigms are generally plagued by limited intermediate supervision. Supervision is typically applied only to the model’s final output, while the intermediate feature fusion process lacks a direct optimization objective. This deficiency in supervision constrains the representational power of multi-level fused features, thereby leading to ambiguity and uncertainty.

Although multispectral semantic understanding methods have demonstrated the value of structured cross-modal interaction, infrared–visible image fusion faces the further challenge of converting such semantic collaboration into task-friendly fused images, which motivates a review of recent task-oriented fusion studies.

### 2.2. Task-Oriented Image Fusion

Earlier deep learning-based infrared–visible image fusion methods primarily emphasized perceptual quality through improved feature extraction and image reconstruction. However, more recent studies have increasingly shifted attention toward semantic completeness and compatibility with downstream tasks. For example, methods based on progressive semantic injection and scene-fidelity preservation [15] show that high-level semantic cues are beneficial for maintaining structural consistency and salient objects. PIAFusion [16] improves perceptual fusion quality through a progressive illumination-aware strategy, while TarDAL [17] explores object-aware adversarial learning for object-detection-oriented fusion. SeAFusion [18] further incorporates semantic-aware optimization into a real-time fusion framework, underscoring the importance of coupling image fusion with downstream perception. Nevertheless, these methods mainly demonstrate the effectiveness of incorporating semantic or task-specific cues into the fusion process, whereas their optimization remains largely centered on improving fusion quality or supporting a specific downstream objective. Consequently, progressive cross-modal correction is only weakly modeled, and the propagation of semantic guidance across stages, as well as its compensation during decoding, is not explicitly addressed.

This direction has been further advanced by recent task-oriented and semantic-guided studies. MetaFusion [19] introduced object-detection-derived meta-feature embedding to guide fusion toward downstream-friendly representations. Liu et al. [20] investigated multi-interactive feature learning and established a benchmark spanning image fusion and segmentation, highlighting the importance of sustained multimodal interaction across tasks. CoCoNet [21] improved multimodal representation quality through coupled contrastive learning and multi-level feature ensemble. Zheng et al. [22] probed synergistic high-order interaction to capture more complex dependencies between infrared and visible modalities. Liu et al. [23] developed a semantic-driven coupled network that explicitly injects semantic cues into the fusion process, while DCEvo [24] further advanced multimodal representation learning through discriminative cross-dimensional evolutionary learning. Complementary to these semantically driven studies, Li et al. [25] addressed infrared–visible image fusion by mitigating modality discrepancy and enhancing spatial consistency. In CSSA-Fusion, cross-modal representations are refined through channel-selective enhancement and redundancy suppression, and fusion quality is further improved via direction-aware feature processing and explicit spatial alignment. However, these methods have largely focused on enhancing individual components of the pipeline, such as task-specific supervision, cross-modal representation interaction, or alignment-aware refinement. As a result, semantic guidance, cross-stage information correction, and decoder-side compensation have generally been optimized in a loosely coupled manner, rather than being incorporated into a unified collaborative framework.

Taken together, these studies indicate that semantic priors, richer multimodal interactions, and tighter coupling with downstream tasks are crucial for high-quality infrared–visible fusion. Nevertheless, a unified framework that jointly performs stage-wise cross-modal correction, progressive semantic-guided interaction, and decoder-side semantic compensation remains insufficiently explored. DSIFuse is developed from this perspective, aiming to improve semantic consistency, detail preservation, and downstream task compatibility within a single collaborative framework.

## 3. Methods

**Figure 2:**
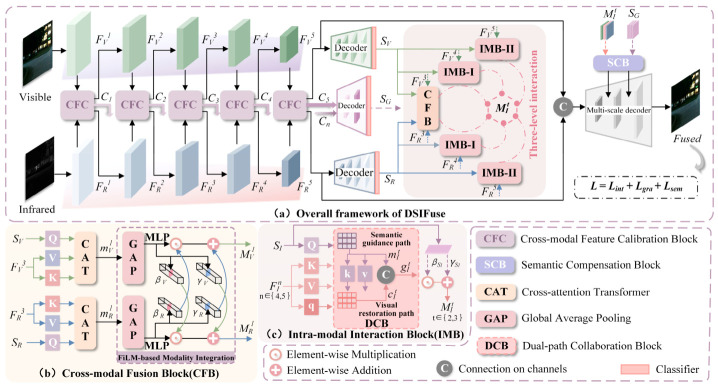
The schematic of the proposed DSIFuse. (**a**) DSIFuse employs a multi-scale encoder with CFC for early cross-modal interaction and feature extraction. Coarse segmentation maps provide semantic priors for subsequent processing. A three-level interaction block performs progressive, semantic-guided feature alignment and fusion. The SCB mitigates semantic degradation before a multi-scale decoder reconstructs the final fused image, optimized by a combined loss; (**b**) CFB uses CAT- and FiLM-based modality integration to fuse features, guided by coarse segmentation maps; (**c**) IMB enhances intra-modal features through semantic guidance and visual restoration paths.

The DSIFuse framework is composed of five stages: visible and infrared encoders, cross-modal feature calibration, tri-level semantic decoder supervision, hierarchical three-level interactive fusion, and multi-scale decoding. The overall network architecture is illustrated in Figure 2.

### 3.1. Framework Overview

Multi-scale hierarchical features are extracted from the visible (V) and infrared (R) modalities using a ResNet-based encoder [26], leveraging its deep residual learning capability. These two branches process modality-specific data in parallel while maintaining interactive information flow. Let $F_{V}^{n}$ and $F_{R}^{n}$ denote the multi-level visible and infrared features obtained from the nth block $\left(n\in \left\{1,2,3,4,5\right\}\right)$ of ResNet, respectively. For modeling multimodal complementarity with long-range dependencies across spatial-channel feature domains, a calibration mechanism (CFC) is integrated between the two branches. Through this mechanism, features from one modality are calibrated using contextual information from the other, enabling effective cross-modal interaction. A stage-wise exchange of the calibrated features between the two modalities promotes comprehensive feature alignment.

The calibrated modality-specific features $F_{I}\;(I\in \left\{V,R\right\})$ and fusion feature $C_{n}\;(n\in \left\{1,2,3,4,5\right\})$ are further exploited by auxiliary segmentation branches to generate semantic guidance maps. Specifically, the deepest calibrated feature in each modality-specific branch is decoded into a coarse segmentation map, denoted as $S_{I}\;(I\in \left\{V,R\right\})$, which is regarded as the semantic prior of the corresponding modality. In parallel, the fusion features ${\left\{C_{n}\right\}}_{n=1}^{5}$ are decoded to produce a global coarse segmentation map $S_{G}$, which captures the shared scene-level semantic context across the two modalities. $S_{I}$ and $S_{G}$ are subsequently used for hierarchical interaction and semantic compensation, respectively. Feature interaction between $F_{I}$ and $S_{I}$ operates at scales n=3,4,5, forming a three-level hierarchy. Following the three-level interaction, a set of hierarchical interaction representations $M_{I}^{t}(t\in \left\{1,2,3\right\})$ is obtained, where t denotes the interaction level. $M_{I}^{t}$ is residually added to the corresponding modality-specific encoder feature. Specifically, $M_{V}^{1}$ and $M_{R}^{1}$ are added to $F_{V}^{3}$ and $F_{R}^{3}$, $M_{V}^{2}$ and $M_{R}^{2}$ are added to $F_{V}^{4}$ and $F_{R}^{4}$, and $M_{V}^{3}$ and $M_{R}^{3}$ are added to $F_{V}^{5}$ and $F_{R}^{5}$, respectively. Furthermore, to mitigate semantic loss during decoder upsampling, $M_{I}^{t}(t\in \left\{1,2\right\})$ and $S_{global}$ are fed into a semantic compensation block (SCB) to generate the compensation feature $A_{f}$. Finally, the fused image ${D_{f}}^{\prime }\in R^{H\times W\times 3}$ is produced by integrating $A_{f}$ with decoder-layer features $D_{f}^{n}(n\in \left\{5,4,3,2,1\right\})$.

**Figure 3:**
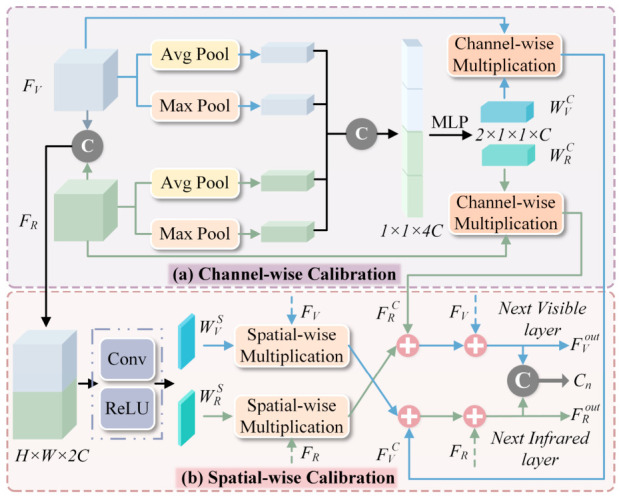
Cross-modal feature calibration block (CFC). (**a**) Channel-wise Calibration: Global average and max pooling are applied to the visible and infrared features, and the resulting descriptors are fed into an MLP to generate modality-specific channel weights, which are then used to recalibrate the complementary modality. (**b**) Spatial-wise Calibration: The bimodal features are concatenated and projected to produce spatial attention maps. The channel- and spatial-calibrated features are finally injected into the opposite streams through residual fusion, yielding bidirectionally calibrated outputs.

Infrared and visible modalities exhibit inherent complementarity. Although each modality inevitably introduces its own noise, its complementary information enables mutual calibration. Building on this insight, the CFC module (Figure 3) operates on the principle of bidirectional calibration, enabling infrared features to suppress noise in visible representations while visible information simultaneously calibrates infrared features. The module is embedded between two adjacent backbone stages, where bidirectionally calibrated features from both modalities are propagated to the subsequent stage for progressive refinement and enhancement of feature extraction.

### 3.2. Cross-Modal Feature Calibration

As described above, infrared and visible modalities provide complementary information, yet each is affected by modality-specific noise. Features from one modality can be exploited to filter and calibrate noisy information from the other. To this end, the proposed cross-modal feature calibration block (CFC) in Figure 3 facilitates bidirectional feature calibration across parallel streams at every feature-extraction stage. For handling cross-modal noise and uncertainty, CFC processes features along both channel and spatial dimensions, enabling holistic feature calibration and thereby enhancing multimodal feature extraction and interaction.

#### 3.2.1. Channel-Wise Calibration

Global average pooling and global max pooling are applied along the spatial dimension to the input features $F_{V}^{n}\in R^{H\times W\times C}$ and $F_{R}^{n}\in R^{H\times W\times C}$, preserving global channel-wise information. The four resulting feature vectors are then concatenated to form $Y\in R^{1\times 1\times 4C}$. Subsequently, a multi-layer perceptron (MLP) followed by a sigmoid activation function is applied to Y, the channel weights $W_{V}^{C}$ and $W_{R}^{C}$ are generated:

(1) $$W_{V}^{C},W_{R}^{C}=F_{split}\left(\sigma \left(F_{mlp}\left(Y\right)\right)\right),$$

where σ(·) denotes the sigmoid activation function. The channel-wise calibration operation is as follows:

(2) $$F_{V,rec}^{C}=W_{R}^{C}⨂F_{R}^{n},F_{R,rec}^{C}=W_{V}^{C}⨂F_{V}^{n}.$$

where ⨂ denotes channel-wise multiplication.

#### 3.2.2. Spatial-Wise Calibration

To further calibrate local information, CFC performs supplementary calibration in the spatial dimension. The inputs $F_{V}^{n}$ and $F_{R}^{n}$ are concatenated and projected into two spatial attention maps: $W_{V}^{S}\in R^{H\times W}$ and $W_{R}^{S}\in R^{H\times W}$. The projection is implemented through two 1 × 1 convolutional layers followed by a ReLU activation. A sigmoid function is then applied to generate the embedded feature map $F\in R^{H\times W\times 2}$, which is subsequently divided into two spatial attention maps. The formulation is as follows:

(3) $$F={Conv}_{1\times 1}\left(RELU\left({Conv}_{1\times 1}\left(F_{V}^{n}∥F_{R}^{n}\right)\right)\right),$$

(4) $$W_{V}^{S},W_{R}^{S}=F_{split}\left(\sigma \left(F\right)\right).$$

Analogous to the channel-wise calibration, the spatial-wise calibration follows an analogous formulation:

(5) $$F_{V,rec}^{S}=W_{R}^{S}⊛F_{R}^{n},F_{R,rec}^{S}=W_{V}^{S}⊛F_{V}^{n}.$$

where ⊛ denotes spatial-wise multiplication. The final dual-modal output features $F_{V}^{out}$ and $F_{R}^{out}$ are obtained through residual fusion:

(6) $$\begin{array}{c} F_{V}^{out}=F_{V}^{n}+\lambda_{C}F_{V,rec}^{C}+\lambda_{S}F_{V,rec}^{S},\\ F_{R}^{out}=F_{R}^{n}+\lambda_{C}F_{R,rec}^{C}+\lambda_{S}F_{R,rec}^{S}. \end{array}$$

where $\lambda_{C}$ and $\lambda_{S}$ are equilibrium coefficients, both set to default values of 0.5. $F_{V}^{out}$ and $F_{R}^{out}$ are comprehensively calibrated features, which will be fed into the subsequent stage. The concatenated features $C_{n}$ serve as the input to the global semantic decoder.

### 3.3. Three-Level Interaction Strategy

#### 3.3.1. Cross-Modal Fusion Block

At the CFB’s first level (t=1), which is applied to the encoder stage n=3, a cross-attention transformer (CAT) and a FiLM-based modality integration block (FMI) are designed. Prior to cross-attention, the semantic priors $S_{I}$ is resized to match the spatial resolution of $F_{I}^{3}$ and flattened along the spatial dimension. Thus, $Q_{I}^{seg}=W_{I}^{q}S_{I}$, ${K_{I}^{seg}=W}_{I}^{k}F_{I}^{3}$, and ${V_{I}^{seg}=W}_{I}^{v}F_{I}^{3}$ each contain $H_{3}W_{3}$ tokens, and $Q_{I}^{seg}{\left(K_{I}^{seg}\right)}^{T}\in R^{H_{3}W_{3}\times H_{3}W_{3}}$. The softmax in Equation (7) is applied along the key dimension for each query token. The resulting interaction feature is then reshaped to the same spatial size as $F_{I}^{3}$ by constructing a cross-attention mapping where $S_{I}$ serves as the query (Q) vector, and $F_{I}^{3}$ provides the key (K) and value (V) vectors, thereby prioritizing visual features aligned with semantic knowledge. This configuration enables the semantic-guided feature fusion, yielding a preliminary interaction feature $m_{I}^{1}$ via the CAT. The interaction is formulated as follows:

(7) $$m_{I}^{1}=softmax\left(\frac{(W_{I}^{q}S_{I})(W_{I}^{k}F_{I}^{3}{)}^{T}}{\sqrt{d_{k}}}\right)\left(W_{I}^{v}F_{I}^{3}\right),$$

where $I\in \left\{V,R\right\}$. W denotes the weight matrix of the linear mapping. $\sqrt{d_{k}}$ is the scaling factor used to stabilize gradients. To mitigate the discrepancies in cross-model feature distribution, a condition-based FMI performs an affine transformation on the primary features $m_{vis}^{1}$ and $m_{inf}^{1}$. This transformation is facilitated by the scaling and offset coefficients (β,γ) generated from the corresponding conditional features via a convolutional layer, yielding the adapted representation $M_{I}^{t}(t=1)$ for reintegration into the corresponding modality branch. The process is defined as follows:

(8) $$M_{I}^{1}=\left(\beta_{I}+1\right)⊙m_{I}^{1}+\gamma_{I},$$

(9) $${\tilde{F}}_{I}^{3}=M_{I}^{1}+F_{I}^{3}.$$

where ⊙ denotes element-wise multiplication.

#### 3.3.2. Intra-Modal Interaction Block

The IMB is structured with two cascaded cross-attention paths at levels t=2 and t=3, corresponding to encoder stages n=4 and n=5, respectively, aiming at balancing semantic prior knowledge and visual details. Specifically, the semantic-guided path integrates global semantic prior knowledge, enabling modal features to be guided and enhanced by high-level semantic information. For the interaction levels $t\in \left\{2,\;3\right\}$, $S_{I}$ is resized according to the spatial size of $F_{I}^{n}\;(n\in \left\{4,\;5\right\})$ and reused to guide the semantic-guided path. Within this path, $S_{I}$ maps to Q, while $F_{I}^{n}$ maps to K and V, whose interaction produces $m_{I}^{t}$. By mapping $F_{I}^{n}$ as the query (q) and $m_{I}^{t}$ as the key (k) and value (v), the visual recovery path compensates for detail loss, producing an interactive representation $c_{I}^{t}$ with enhanced visual fidelity. Concatenating the outputs $m_{I}^{t}$ and $c_{I}^{t}$ from both paths yields a fine-grained representation $g_{I}^{t}$ that balances semantic and detailed information. Subsequently, $g_{I}^{t}$ is adaptively modulated by (β,γ), which are conditioned on $S_{I}$ in the FMI block, thereby enhancing the completeness of the final output. Finally, the resulting $M_{I}^{t}$ is fed back into the encoder layer. The computations for $M_{I}^{t}$ and $F_{I}^{n}$ are as follows:

(10) $$M_{I}^{t}=\left(\beta_{S_{I}}+1\right)⊙g_{I}^{t}+\gamma_{S_{I}},$$

(11) $${\tilde{F}}_{I}^{n}=M_{I}^{t}+F_{I}^{n}.$$

where $\left(\beta_{S_{I}},\;\gamma_{S_{I}}\right)$ denotes the scaling and shift coefficients for $S_{I}$.

### 3.4. Semantic Compensation Block

**Figure 4:**
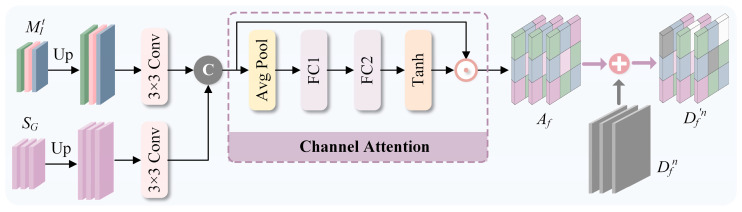
Framework of the semantic compensation block (SCB).

To mitigate the semantic loss induced by upsampling in the decoder, $M_{I}^{t}\;(t\in \left\{1,\;2\right\})$ and $S_{G}$ are compensated into their respective decoder layers via SCB, as detailed in Figure 4. Specifically, $M_{I}^{t}$ and $S_{G}$ are upsampled separately to align their spatial resolutions with the object feature $D_{f}^{n}$ to be calibrated. Subsequently, these upsampled features undergo a 3 × 3 convolution followed by concatenation. Channel attention is then applied to the concatenated features to obtain $A_{f}$. Finally, $A_{f}$ is element-wise added to $D_{f}^{n}\;(n\in \left\{4,5\right\})$ to yield ${D_{f}^{n}}^{\prime }$, which then continues through the subsequent decoding process.

### 3.5. Fusion Loss

To attain a holistic and coherent fusion feature, a composite loss function L is designed, comprising intensity loss $L_{int}$, gradient loss $L_{gra}$, and semantic prior loss $L_{sem}$. L is defined as follows:

(12) $$L=\mu L_{int}+\zeta L_{gra}+\eta L_{sem}.$$

where μ, ζ, and η denote the weighting coefficients for $L_{int}$, $L_{gra}$, $L_{sem}$, respectively. Inspired by [15], a salient object mask $M_{label}$, derived from the dataset-provided binary annotation mask, is introduced to guide the fusion network for preserving salient infrared targets. Specifically, for each training pair, pixels belonging to the annotated foreground target are assigned a value of 1, whereas background pixels are assigned 0, yielding $M_{label}\in {\left\{0,1\right\}}^{H\times W}$. $L_{int}$ is expressed as follows:

(13) $$\begin{array}{c} L_{int}=\frac{1}{HW}{∥\delta \left(M_{R}+M_{label}\right)⊙\left(D_{f}^{\prime }-X_{R}\right)∥}_{1}+\\ \;\;\;\;\;\;\;\;\;\;\;\;\;\;\;\;\;\;\;\;\;\frac{1}{HW}{∥\delta \left(M_{V}+\left(1-M_{label}\right)\right)⊙\left(D_{f}^{\prime }-X_{V}\right)∥}_{1}, \end{array}$$

(14) $$M_{R}=Norm\left({Var}_{k\times k}\left(X_{R}\right)\right),\;M_{V}=Norm\left({Var}_{k\times k}\left(X_{V}\right)\right),$$

where δ(x) denotes a binary thresholding operator applied to the combined masks, which outputs 1 for positive inputs and 0 otherwise. Norm(·) denotes min–max normalization, which rescales the variance map to the range [0, 1]. ${Var}_{k\times k}$ denotes the pixel-wise local variance computed within a k × k neighborhood, where k is the side length of the local window and is set to 3 in all experiments. $M_{R}$ and $M_{V}$ serve as contrast-aware soft masks, where larger values indicate regions with richer local structures. $D_{f}^{\prime }$ is the final fused image. $X_{R}$ and $X_{V}$ denote the source infrared and visible images, respectively. ${∥\cdot ∥}_{1}$ denotes the L1 norm.

Additionally, the $L_{gra}$ leverages a max-selection rule on the source gradients to preserve critical texture details, as formulated below:

(15) $$L_{gra}=\frac{1}{HW}{∥\left|\nabla D_{f}^{\prime }\right|-max\left(\left|\nabla X_{R}\right|,\left|\nabla X_{V}\right|\right)∥}_{1},$$

where ∇ is the Sobel gradient operator, and $\left|\cdot \right|$ denotes the absolute value. To constrain prior knowledge learning, a semantic prior loss $L_{sem}$ is constructed to ensure the fused image retains complete semantic information during the three-stage interaction. Incorporating multi-scale intermediate features into the loss calculation provides deeper supervision for extracting beneficial information. $L_{sem}$ is defined as follows:

(16) $$L_{sem}=\sum_{t=1}^{3}\left\{\frac{1}{H_{t}W_{t}}\left[{\left(S_{R}^{t}-E^{t}\right)}^{2}+{\left(S_{V}^{t}-E^{t}\right)}^{2}\right]\right\}.$$

where $S_{R}^{t}$ and $S_{V}^{t}$ represent the semantic priors at level t, generated by upsampling or downsampling $S_{R}$ and $S_{V}$. $E^{t}$ denotes the multi-scale semantic ground-truth map obtained by resizing the pixel-wise annotation map to the spatial resolution $H_{t}\times W_{t}$. Nearest-neighbor interpolation is adopted to preserve discrete semantic boundaries. Since each visible–infrared pair depicts the same scene, the shared $E^{t}$ is used to supervise both $S_{R}^{t}$ and $S_{V}^{t}$. These semantic ground-truth maps are only used during training. $H_{t}$ and $W_{t}$ denote the height and width of $S_{I}^{t}$ at level t.

## 4. Experiments

This section first introduces the implementation details and configuration of the DSIFuse network. Subsequently, extensive experiments on multiple public datasets are conducted to evaluate the model’s performance. The superiority of DSIFuse is demonstrated by comparing its fusion performance against various state-of-the-art algorithms from qualitative and quantitative perspectives, encompassing image fusion evaluation metrics, downstream object detection accuracy, and operational efficiency. Finally, ablation studies are conducted to isolate and highlight the contributions of each design component within the model architecture to the overall performance improvement.

### 4.1. Experimental Protocol

#### 4.1.1. Datasets

To assess the fusion performance of DSIFuse, comprehensive experiments were conducted utilizing three publicly available datasets: MSRS [16], M3FD [17], and TNO [27]. The DSIFuse network was trained on the MSRS training set (1083 pairs), while the MSRS test set (361 pairs), the M3FD image fusion dataset (300 pairs), and the TNO (37 pairs) were served as test sets to comprehensively validate fusion performance. Additionally, further evaluation of fusion image quality was conducted on the M3FD object detection dataset (4200 pairs).

#### 4.1.2. Comparison Methods

To demonstrate the superiority of DSIFuse, nine widely used fusion methods were selected for comparison. These include three autoencoder-based methods: DenseFuse [28], DRF [29], and MFIFusion [30]; three convolutional neural network-based methods: SeAFusion [18], UMF-CMGR [31], and LRRNet [32]; one generative adversarial network-based method: FusionGAN [33]; and two Transformer-based methods: CDDFuse [34] and CrossFuse [35].

#### 4.1.3. Evaluation Metrics

A high-quality fusion image should effectively capture salient objects and preserve visual quality from multimodal images. To comprehensively assess fusion outcomes, we employ six metrics for quantitative evaluation: information–theoretic measures including entropy (EN) [36] and mutual information (MI) [37]; feature-based metrics such as average gradient (AG) [38] and standard deviation (SD) [39]; human-perception-based visual information fidelity (VIF) [40] and structural similarity-based structural similarity measure (SSIM) [41]. Higher metric values indicate superior fusion results. Additionally, the standard evaluation method using mean average precision (mAP) validated the effectiveness of the DSIFuse in object localization and detection tasks. Here, mAP_50_ denotes the mean AP at an IoU threshold of 0.5, while mAP_50:95_ denotes the average AP over IoU thresholds from 0.50 to 0.95 with a step size of 0.05. Formally,

(17) $${mAP}_{50}=\frac{1}{C}\sum_{C=1}^{C}{AP}_{C}^{0.5},$$

(18) $${mAP}_{50:95}=\frac{1}{10C}\sum_{t=0}^{9}\sum_{C=1}^{C}{AP}_{C}^{0.50+0.05t}.$$

where C is the number of object categories, and ${AP}^{\tau }$ denotes the average precision of category C at IoU threshold τ. IoU denotes the intersection over union between the predicted bounding box and the ground-truth bounding box.

#### 4.1.4. Training Details

Our DSIFuse was trained on the PyTorch 1.13 platform under the following empirical configuration: 1900 epochs, a batch size of 16, and loss weighting coefficients set to μ = 155, ζ = 10, and η = 7. These coefficients were determined by grid search on a validation subset of the training set, and the final setting was adopted because it achieved the best balance among intensity fidelity, gradient preservation, and semantic consistency. The initial learning rate was set as 0.001, and the poly-learning rate decay strategy was employed to effectively extract semantic information. All experiments were conducted on an NVIDIA GeForce RTX 5070Ti GPU (NVIDIA Corp., Santa Clara, CA, USA) and an AMD Ryzen 7 9700X 8-Core Processor CPU (Advanced Micro Devices, Inc., Santa Clara, CA, USA).

### 4.2. Fusion Comparison and Analysis

#### 4.2.1. Results of Infrared–Visible Image Fusion

**Figure 5:**
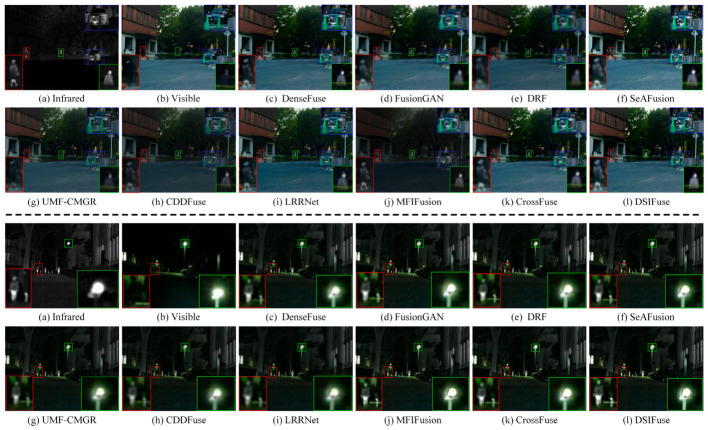
Qualitative comparison of DSIFuse with 9 state-of-the-art approaches on the “00127D” and “01028N” images from the MSRS dataset. Color rectangles represent highlighted details.

**Figure 6:**
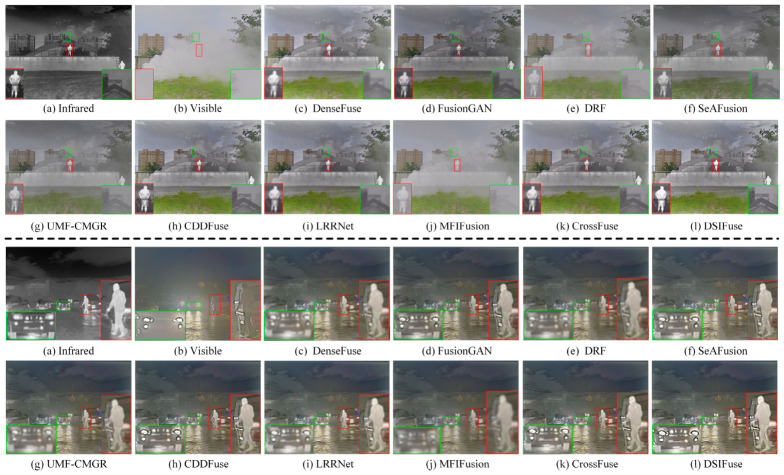
Qualitative comparison of DSIFuse with 9 state-of-the-art approaches on the “00409” and “00916” images from the M3FD dataset. Color rectangles represent highlighted details.

**Figure 7:**
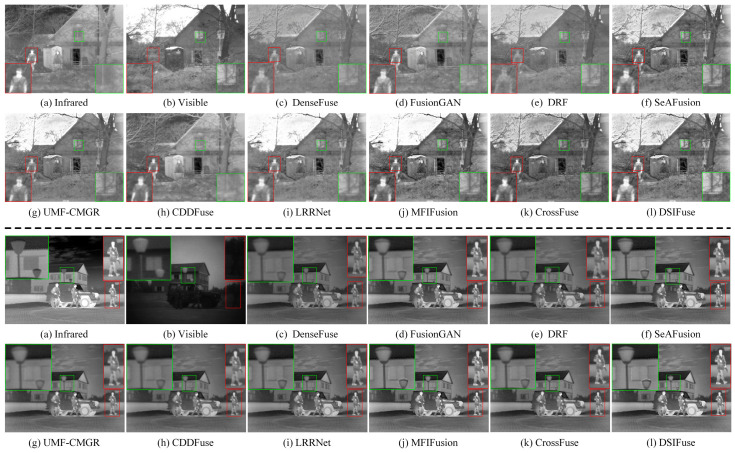
Qualitative comparison of DSIFuse with 9 state-of-the-art approaches on the “001” and “011” images from the TNO dataset. Color rectangles represent highlighted details.

(1) Qualitative Comparison and Analysis: Figure 5, Figure 6 and Figure 7 present qualitative visualization results from six representative image pairs sourced from the MSRS, M3FD, and TNO datasets. For clearer comparison, prominent object regions are annotated (indicated by red, green, and blue rectangles) and displayed in enlarged views. Figure 5 demonstrates that across two representative MSRS scenes, pedestrians in dark areas of our DSIFuse-fused image exhibit high contrast and sharp contours, while effectively preserving vehicle texture details. Moreover, under challenging nighttime conditions involving weak objects and bright light sources, DSIFuse effectively suppresses overexposure and preserves local structural details, thereby maintaining both object saliency and structural fidelity. In contrast, visual inspection reveals that FusionGAN, DRF, and UMF-CMGR fail to effectively highlight discriminative objects, while LRRNet and CDDFuse cannot preserve rich texture details. Although SeAFusion and MFIFusion produce distinct objects with well-preserved texture details, their contrast and clarity fall short of our approach. Overall, superior fusion quality is achieved by DSIFuse, as evidenced by clearer salient objects, sharper boundaries, and more faithful texture preservation under varying illumination conditions. This advantage stems from three key aspects. First, multi-level cross-modal interactions and intra-modal enhancement mechanisms ensure effective extraction, precise alignment, and deep fusion of distinct modal features. Furthermore, the semantic compensation block and global semantic priors were devised to guide the fusion process, maintaining high semantic consistency while compensating for and refining potentially missing details in the fusion results. This simultaneously highlights prominent objects and preserves fine textures. Finally, a refined loss function based on contrast masks and salient object masks was designed to maintain the visual appeal of the fused images. As shown in Figure 6, under challenging visibility conditions, most fusion algorithms fail to produce satisfactory fusion results. In the smoke-filled scene, buildings obscured by smoke cannot be clearly recovered by most methods, despite the preservation of salient infrared objects. In the low-illumination scene with strong glare, competing methods tend to suffer from object blurring and loss of local details in bright regions, whereas DSIFuse preserves clearer pedestrian contours and richer structural information.

Additionally, qualitative results on the TNO dataset further verify the superior fusion performance of DSIFuse. As shown in Figure 7l, the salient human objects in the infrared image and the fine structural details in the visible image, such as the branches near the window, are simultaneously preserved. Thermal radiation cues and visible-texture information are effectively integrated, thereby avoiding the distortion and overexposure observed in several competing methods. Specifically, the soldier’s shape and contours are accurately retained, while the branches and background structures remain clearly discernible without excessive blurring or information loss. A similar trend is observed in the other TNO scene, where the thermal saliency of the human figures is retained, and the structural details of the lamp post, roofline, and house façade in the highlighted regions are more faithfully reconstructed. By contrast, several competing methods suffer from object diffusion, reduced contrast, or structural blurring, which degrades both salient-object representation and scene-detail fidelity. These results indicate that DSIFuse achieves a better balance between semantic saliency and fine-detail preservation than other representative methods, especially in complex backgrounds and around prominent objects. Through the proposed multi-level collaborative learning strategy, semantic information and visual details are effectively coordinated, ensuring clearer and more reliable fusion results. Extensive qualitative comparisons further confirm the perceptual superiority of DSIFuse under challenging conditions, including nighttime, fog, occlusion, and glare, thereby better supporting downstream tasks.

(2) Quantitative Comparison and Analysis: Table 1, Table 2 and Table 3 present the comparison results of multiple average quantitative evaluation metrics between DSIFuse and other mainstream fusion methods across three datasets. Among these metrics, AG, SD, VIF, and SSIM consistently exhibit superior values, further confirming that the fusion results generated by DSIFuse achieve excellent structural consistency and high perceptual quality. Notably, MFIFusion outperforms DSIFuse in EN values across all three datasets, indicating that MFIFusion’s fusion images contain richer information content. However, higher EN does not necessarily imply superior fusion quality. The elevated EN values of MFIFusion primarily arise from its pixel-level multi-scale feature superposition strategy, which expands grayscale distribution and enhances local textures. While this approach excels in information density, the lack of high-level semantic constraints may introduce redundant information and local texture fluctuations. For instance, the green-boxed area in Figure 7j exhibits poor contrast and reduced clarity in windows and branches, along with unnecessary texture fluctuations. Similarly, the red-boxed region fails to preserve detailed human textures effectively, resulting in blurred contours and degraded structural integrity.

Although DSIFuse exhibits slight declines in EN and MI metrics on MSRS and M3FD datasets, it maintains high performance levels. Through multi-level semantic fusion and attention mechanisms, DSIFuse emphasizes image structural fidelity and fine-detail preservation. While this strategy slightly lowers information entropy and mutual information, it significantly enhances visual quality, detail preservation, and structural consistency. In summary, DSIFuse stably preserves essential information from the source images, effectively retaining the most discriminative objects, rich textural details, and high-fidelity structural similarity.

#### 4.2.2. Results of Infrared–Visible Object Detection

To thoroughly assess the impact of DSIFuse on downstream task performance, object detection experiments were conducted on the fusion results using YOLOv8 [42]. These experiments were performed on the M3FD dataset to assess the effects of different fusion algorithms on object detection tasks.

**Figure 8:**
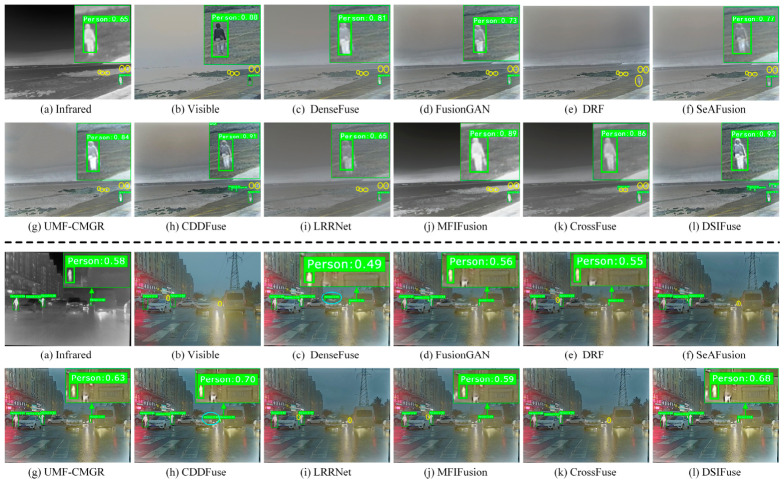
Detection results of DSIFuse with 9 state-of-the-art approaches on the “00011” and “01911” images from the M3FD dataset. Cyan and yellow circles indicate false positives and false negatives, respectively.

**Figure 9:**
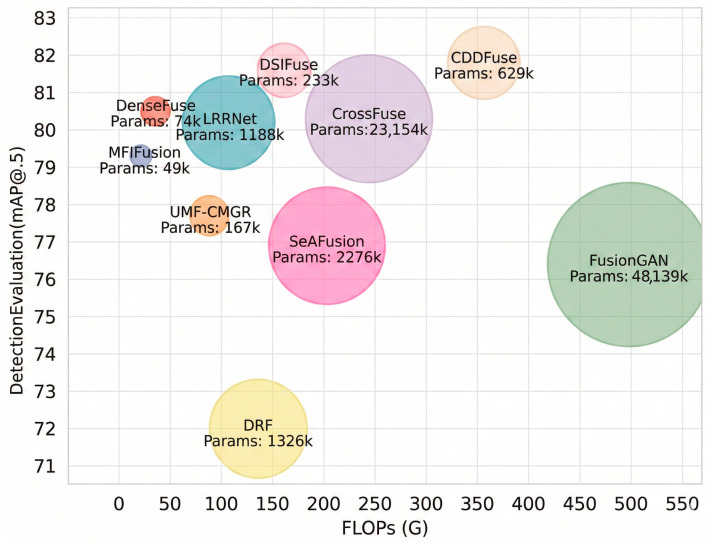
Performance vs. FLOPs on M3FD. DSIFuse shows a highly competitive performance-efficiency trade-off among the state-of-the-art methods.

(1) Qualitative Comparison and Analysis: As seen in Figure 8, a single sensor (infrared or visible) is unable to effectively detect objects. Infrared images exhibit high contrast and allow distant objects to be clearly visualized, but lack fine texture details. In contrast, visible images reveal richer texture and color information, yet perform poorly in low-light and long-range conditions, lacking the contrast advantage of infrared images. By leveraging complementary information from both modalities, nearly all fusion methods enhance detection performance. However, their fusion results still exhibit numerous issues. In the upper scene, SeAFusion, FusionGAN, and DRF tend to over-smooth object and background regions, thereby weakening the discriminability of the distant pedestrian. In the lower rainy scene, DenseFuse and CDDFuse enhance local responses, but distracting activations are also introduced around traffic structures. By comparison, a more semantically coherent representation is produced by DSIFuse: the primary pedestrian in the upper scene is preserved with clearer contours, while a better balance is achieved among distant pedestrian cues, vehicle structures, and rainy background details in the lower scene. Consequently, fewer erroneous detections are produced overall. Nevertheless, a limitation of DSIFuse should also be noted. For several objects, the detection confidence remains slightly lower than that of CDDFuse, indicating that the discriminability of certain local object features has not yet been fully optimized. This observation is consistent with the quantitative results, in which CDDFuse performs marginally better at mAP@0.5, whereas DSIFuse exhibits greater robustness at higher IoU thresholds.
(2) Quantitative Comparison and Analysis: Table 4 presents the quantitative detection results on the M3FD dataset. Nearly all fusion methods achieved improved detection performance, with mAP values surpassing those obtained using only visible or infrared images. DSIFuse ranked first in human, car, and motorcycle detection, remaining in the top-three positions even with slight declines in bus, streetlight, and truck detection. Under the mAP_50_, CDDFuse performed marginally better than DSIFuse. However, DSIFuse exhibited greater stability as detection difficulty increased. Overall, DSIFuse achieves superior performance across multiple categories and metrics, demonstrating particular robustness at high IoU thresholds. This validates its capacity to preserve clear details and deliver sufficient semantic information, culminating in enhanced object detection performance.
(3) Computational Complexity Comparison and Analysis: To comprehensively evaluate DSIFuse’s performance, the FLOPs and training parameters of all compared methods were analyzed. As shown in Figure 9, more complex models (e.g., LRRNet and CrossFuse) showed superior performance but incurred higher computational costs. In contrast, simpler models (e.g., UMF-CMGR and MFIFusion) exhibited lower computational complexity but delivered relatively weaker detection performance. However, DSIFuse maintained a balance between computational efficiency and performance in terms of FLOPs and training parameters. Although its performance was slightly lower than the optimal CDDFuse, DSIFuse achieved comparable performance at a significantly lower computational cost, demonstrating its suitability for deployment in resource-constrained applications.

Additionally, despite DSIFuse achieving lower parameters and ascended computational efficiency, its performance may still be constrained compared with more complex models such as FusionGAN and CrossFuse. When processing scenes with intricate structures or high-detail requirements, these models, owing to their greater parameter capacity, are capable of capturing finer textures and accurately delineating object boundaries, thereby attaining superior performance.

### 4.3. Ablation Studies and Discussion

To further validate the effectiveness of the designed individual components in image fusion, ablation studies were conducted on the MSRS and TNO datasets (w/o- CFC, w/o- CFB, w/o- IMB, w/o- SCB, w/o- S_G_).

1. Quantitative Comparison and Analysis: Table 5 presents the quantitative ablation results of DSIFuse on the MSRS dataset. The complete model achieves the best performance on five of the six metrics, demonstrating the effectiveness of the overall architecture. Among all components, CFC has the largest impact: once it is removed, the most severe performance degradation is observed across all metrics, highlighting its critical role in early cross-modal feature calibration. Performance is also consistently degraded when CFB or IMB is omitted, indicating that both cross-modal fusion and intra-modal interaction are essential for effective feature aggregation. By contrast, removing SCB or SG leads to relatively smaller yet still noticeable declines, suggesting that semantic compensation and semantic guidance provide complementary benefits for detail preservation and structural consistency. Overall, these results confirm that each component contributes positively to the final performance, while the complete model achieves the best overall balance.

**Figure 10:**
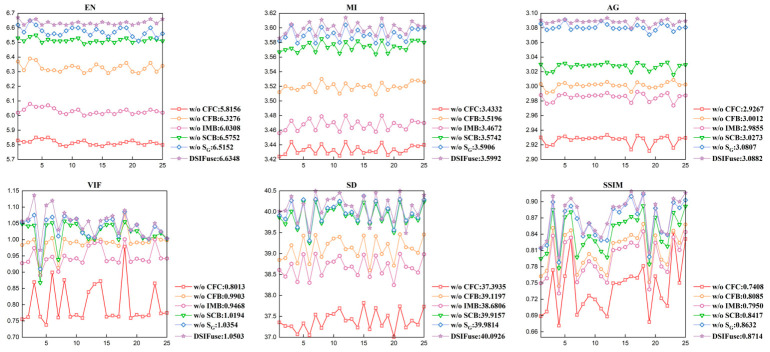
Quantitative comparisons of the six metrics, i.e., EN, MI, AG, SD, VIF, and SSIM, on 25 image pairs from the MSRS database.

To further validate these observations at the sample level, Figure 10 visualizes the quantitative ablation results on 25 randomly selected image pairs from the MSRS dataset using six image-quality metrics. The X-axis represents fusion images generated from 25 randomly selected image pairs in the MSRS dataset, and the Y-axis shows metric values. The legend indicates the average values for each configuration. (1) W/o CFC (red square line) performed worst across all six metrics, typically showing significant gaps compared to the full model and the most pronounced fluctuations. This indicates CFC is the most critical component in the entire model, responsible for effective cross-modal feature alignment and complementary information extraction. Removing CFC caused comprehensive degradation in information content, source information retention, edge sharpness, visual quality, contrast, and structural similarity. (2) Performance degradation is also pronounced for w/o IMB (pink pentagonal line) and w/o CFB (orange circular line). IMB enhances intra-modal features; its absence prevents optimal extraction of modality-specific advantages, compromising final fusion quality. CFB serves as the core fusion mechanism for the deep integration of cross-modal information. Its removal disrupts effective information collaboration, significantly degrading fusion results. (3) Performance degradation for w/o SCB (green triangle line) and w/o S_G_ (blue diamond line) is relatively smaller, yet a consistent decline trend remains observable. While not critical for fundamental information transfer, they play vital roles in enhancing semantic consistency, detail compensation, and overall robustness of the final fusion results. Although w/o S_G_ exhibits a relatively minor decline in quantitative metrics, combined with the previous qualitative analysis (overexposure issues), it uniquely and significantly impacts overall visual quality and brightness control. This suggests its contribution may not be fully reflected in these generic metrics but instead emphasizes visual harmony derived from high-level semantic guidance. (4) DSIFuse (purple star-shaped line) consistently achieved the best performance, with its curve positioned above all others and the highest average values. This confirms that DSIFuse attains optimal image fusion performance through the synergistic integration of all designed modules, outperforming all ablation variants across multiple dimensions, including EN, MI, AG, VIF, SD, and SSIM.

**Figure 11:**
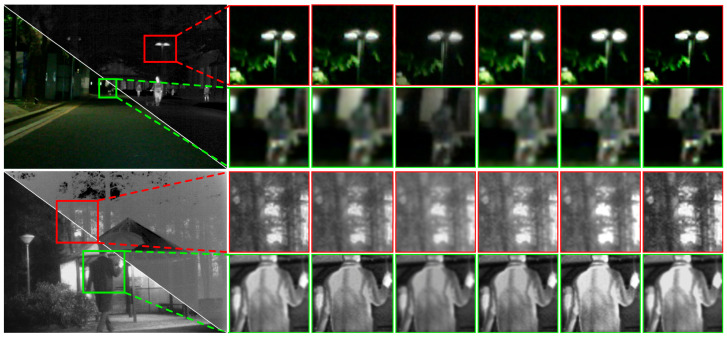
Visualization results of ablation studies on the MSRS and TNO datasets. From left to right: source images, w/o CFC, w/o CFB, w/o IMB, w/o SCB, w/o S_G_, and the full model.

2. Qualitative Comparison and Analysis: As shown in Figure 11, (1) the absence of CFC leads to significant performance degradation, primarily manifested as detail loss and object blurring. This highlights the critical role of effective cross-modal feature calibration. (2) CFB facilitates deep collaborative interaction and integration of multimodal information, serving as the core component for synergistic information enhancement. Its absence directly weakens the multimodal integration mechanism, causing degradation in detail preservation. (3) Without IMB, fusion outputs lack sharpness and clarity, as raw information from each modality remains underutilized. (4) SCB and SG provide semantic consistency and detail compensation. Their absence causes relatively subtle degradation, primarily manifesting in the fineness of semantic boundaries, smoothness within objects, and overall semantic robustness. For example, in the first group (w/o SCB and w/o SG), the brightness of the streetlight area within the red box is notably higher than in the complete model. The surrounding leaf details also become blurred due to overexposure, losing the clarity and color gradation seen in the full model. This suggests that without global semantic guidance, the network fails to effectively regulate light source brightness output. At a deeper level, semantic guidance is crucial for the network’s understanding and control of overall image visual quality and intensity distribution. Its absence weakens the network’s ability to adaptively adjust high-intensity information based on the scene’s semantic context, leading to image saturation and overexposure. (5) The full model consistently delivers optimal performance. DSIFuse synergistically leverages the strengths of all modules, effectively integrating the rich details of visible images with the strong semantic information from thermal images. The fusion outputs achieve an optimal balance in detail, semantic representation, and clarity. This fully validates the rationality and effectiveness of each DSIFuse block.

## 5. Conclusions

This study tackles the challenges of semantic misalignment and detail degradation in multi-modal image fusion, focusing on achieving effective cross-modal feature interaction under semantic guidance. We demonstrate that explicit semantic supervision and progressive feature integration are crucial for generating fusion results that concurrently cater to human visual perception and machine vision tasks. Based on this insight, we propose a semantic-guided image fusion network called DSIFuse. The network initiates with a cross-modal feature calibration to align different modalities. Following this, an intra-modal interaction block progressively refines features within each modality, while a cross-modal fusion block facilitates deep information interaction across modalities. To maintain contextual consistency and restore fine details, a semantic compensation is further incorporated. Furthermore, a tailored fusion loss is constructed to optimize image quality and semantic objectives. Comprehensive quantitative and qualitative evaluations demonstrate that DSIFuse outperforms state-of-the-art image fusion algorithms in visual quality and semantic consistency. More importantly, the fused outputs were shown to achieve competitive detection performance on the YOLOv8-based M3FD benchmark, especially at higher IoU thresholds. Furthermore, the effectiveness and necessity of our network’s core components are substantiated through ablation analyses. In terms of computational efficiency, DSIFuse attains superior performance with a lightweight architecture, striking an excellent balance between fusion accuracy and runtime speed that renders it advantageous for real-time and embedded applications. Looking ahead, we will focus on integrating temporal information to extend the framework for video fusion, addressing temporal coherence and real-time demands to support robotics, surveillance, and AR.

## Figures and Tables

**Table 1:** Quantitative comparisons of selected schemes on 361 pairs of images in the MSRS dataset. Red indicates the best result. Blue indicates the second-best result. “↑” indicates that for these image fusion evaluation metrics, higher values are better.

Methods	EN ↑	MI ↑	AG ↑	SD ↑	VIF ↑	SSIM ↑
DenseFuse [28]	5.937	2.643	2.058	23.568	0.692	0.911
FusionGAN [33]	5.432	1.886	1.451	17.07	0.443	0.502
DRF [29]	5.769	2.069	1.421	20.991	0.494	0.470
SeAFusion [18]	6.658	3.226	2.584	35.375	0.704	0.904
UMF-CMGR [31]	5.597	1.920	2.134	20.745	0.427	0.538
CDDFuse [34]	5.942	3.244	2.397	29.683	0.752	0.655
LRRNet [32]	5.945	3.603	2.405	29.725	0.755	0.905
MFIFusion [30]	7.120	2.558	2.005	26.342	0.636	0.797
CrossFuse [35]	5.900	2.42	1.918	27.521	0.757	0.833
Ours	6.636	3.596	3.089	39.972	1.050	0.915

**Table 2:** Quantitative comparisons of selected schemes on 300 pairs of images in the M3FD dataset. Red indicates the best result. Blue indicates the second-best result. “↑” indicates that for these image fusion evaluation metrics, higher values are better.

Methods	EN ↑	MI ↑	AG ↑	SD ↑	VIF ↑	SSIM ↑
DenseFuse [28]	6.796	2.930	3.303	32.398	0.762	0.868
FusionGAN [33]	6.513	2.560	2.679	27.160	0.656	0.759
DRF [29]	6.758	2.688	2.876	30.649	0.771	0.675
SeAFusion [18]	6.887	2.765	3.697	33.352	0.706	0.902
UMF-CMGR [31]	6.734	2.236	2.143	24.467	0.568	0.855
CDDFuse [34]	5.772	3.235	2.378	30.873	0.712	0.776
LRRNet [32]	6.372	3.364	2.563	29.496	0.768	0.865
MFIFusion [30]	6.978	2.684	3.564	26.487	0.725	0.767
CrossFuse [35]	6.834	3.263	3.383	27.865	0.783	0.822
Ours	6.866	3.353	4.210	33.346	0.791	0.911

**Table 3:** Quantitative comparisons of selected schemes on 37 pairs of images in the TNO dataset. Red indicates the best result. Blue indicates the second-best result. “↑” indicates that for these image fusion evaluation metrics, higher values are better.

Methods	EN ↑	MI ↑	AG ↑	SD ↑	VIF ↑	SSIM ↑
DenseFuse [28]	6.181	2.133	2.255	22.568	0.610	0.819
FusionGAN [33]	6.461	2.356	2.352	25.368	0.422	0.679
DRF [29]	6.627	1.932	2.341	29.085	0.323	0.516
SeAFusion [18]	6.896	2.658	4.683	39.556	0.595	0.909
UMF-CMGR [31]	6.307	2.177	2.684	26.274	0.533	0.832
CDDFuse [34]	6.842	2.569	4.331	34.565	0.611	0.520
LRRNet [32]	6.836	2.613	3.716	35.576	0.532	0.866
MFIFusion [30]	6.913	2.332	2.456	30.089	0.505	0.834
CrossFuse [35]	6.835	2.203	3.813	37.992	0.622	0.872
Ours	6.893	2.779	4.732	39.263	0.629	0.916

**Table 4:** Quantitative results of object detection based on various fusion approaches on the M3FD dataset. Red indicates the best result. Blue indicates the second-best result.

Methods	Per	Car	Bus	Mot	Lam	Tru	mAP_50_	mAP_50:95_
Visible	61.5	81.2	88.6	62.1	83.9	72.8	71.9	52.6
Infrared	63.7	73.6	83.4	60.9	86.6	65.2	68.3	48.9
DenseFuse [28]	66.1	88.3	95.2	69.6	89.7	73.0	80.5	53.3
FusionGAN [33]	64.2	85.4	88.8	69.2	89.5	69.4	76.8	52.7
DRF [29]	63.3	81.8	89.7	66.5	84.8	67.3	72.1	51.9
SeAFusion [18]	64.6	83.5	91.1	67.6	88.7	72.7	77.0	52.8
UMF-CMGR [31]	66.8	84.7	92.3	68.8	89.8	72.2	77.9	53.2
CDDFuse [34]	66.5	86.5	95.8	71.8	89.5	71.5	81.8	53.9
LRRNet [32]	64.7	85.1	90.9	69.4	86.2	73.3	80.2	52.6
MFIFusion [30]	64.8	87.7	92.3	67.5	89.3	73.5	79.3	53.6
CrossFuse [35]	65.5	87.9	93.9	70.7	91.3	72.2	80.3	54.2
Ours	67.3	88.6	94.2	72.3	91.0	73.1	81.6	54.8

**Table 5:** Ablation study of DSIFuse on the MSRS dataset. Each ablation removes one component from the full model. Red indicates the best result. “↑” indicates that for these image fusion evaluation metrics, higher values are better.

Methods	EN ↑	MI ↑	AG ↑	SD ↑	VIF ↑	SSIM ↑
w/o CFC	5.813	3.431	2.928	37.394	0.798	0.741
w/o CFB	6.323	3.520	3.003	39.120	0.990	0.809
w/o IMB	6.026	3.465	2.994	38.681	0.947	0.795
w/o SCB	6.575	3.572	3.027	39.912	1.019	0.842
w/o S_G_	6.510	3.591	3.081	39.970	1.035	0.865
Ours	6.636	3.596	3.089	39.972	1.050	0.915

## Data Availability

Data are contained within this article.
